# Evaluating the Diagnostic Agreement between Telepsychiatry Assessment and Face-to-Face Visit: A Preliminary Study 

**Published:** 2019-07

**Authors:** Shahrzad Mazhari, Alireza Ghaffari Nejad, Omid Mofakhami, Farzaneh Raaii, Kambiz Bahaadinbeigy

**Affiliations:** 1 Neuroscience Research Center, Institute of Neuropharmacology, Kerman University of Medical Sciences, Kerman, Iran.; 2 Neurology Research Center, Kerman University of Medical Sciences, Kerman, Iran.; 3 Department of Psychiatry, Shahid-Beheshti Hospital, Kerman University of Medical Sciences, Kerman, Iran.; 4 Medical Informatics Research Center, Institute for Future Studies in Health, Kerman University of Medical Sciences, Kerman, Iran.

**Keywords:** *Diagnosis Agreement*, *Implementation*, *Iran*, *Telepsychiatry*, *Telemental Health*

## Abstract

**Objective:** Despite accumulated evidence that demonstrates clinical outcome of telepsychiatry is comparable with conventional method; little research has been done on telepsychiatry in developing countries. This study aimed to evaluate the diagnostic agreement between telepsychiatry assessment and face-to-face assessment. Moreover, patient and doctor satisfaction was assessed by self-report questionnaire.

**Method** **:** This study was conducted in an inpatient department of a university-affiliated hospital in Kerman University of Medical Sciences, Iran. The study sample consisted of 40 inpatients aged over 18 years who were selected from October 2016 to February 2017. All patients were visited onc

e by face-to-face conventional method and once by interactive video teleconsultation by 2 psychiatric consultants.

**Results: **Results of this study revealed that the diagnostic agreement between the 2 interviewers was 75%. Moreover, about 85% of the patients preferred telepsychiatry for follow-up visits. Also, more than 82% of the patients would recommend telepsychiatry to others although 95% of them perceived contact via telepsychiatry as uncomfortable to some extent.

**Conclusion: **Telepsychiatry service can be used for psychiatric evaluation in Iran, and it has a desirable effect on patient and doctor satisfaction. The results of this study showed the capacity of moving towards using telepsychiatry.

Telemedicine is the use of telecommunications technology for the delivery of health services when the patient and the professional are at distant locations. Telepsychiatry is a branch of telemedicine, which can be defined as the delivery of psychiatric care and exchange of health care information from a central site to a distant or remote area (1).Telepsychiatry usually refers to a 2-way interactive communication (e.g., videoconferencing) for diagnosis, education, and treatment. Videoconferencing is the earliest method and requires the use of studios, which are usually located at hospitals. Developments of new technologies allows for easier interactions using personal computers, tablets, and mobile phones (2).

Several published reviews have reported that telepsychiatry can be helpful in diagnosis, treatment, and follow-up of patients with mental health problems, and it is comparable to in person care (3). Studies have found moderate to high level of agreement between telepsychiatry and face-to-face interview (3, 4). For example, Schutte et al (2015) showed substantial agreement between telepsychiatry and face-to-face assessments (interclass coefficient = 0.92) (5). 

Although telepsychiatry has been successful and widely used in developed countries, it is not commonly investigated in developing countries. Some potential barriers in developing countries are underdeveloped infrastructure, poor internet penetration, poor connectivity, and high costs. In addition, most people living in distant areas are not capable of using computers and the related technology. Finally, the absence of national government support and policies restricts research in these areas. 

Iran is a developing country, where the prevalence of psychiatric disorders is about 22% and psychiatric disorders rank second on the list of burden of disease after unintentional accidents(6). Moreover, prevalence studies have shown that 22.3% of Iranian children and adolescents have at least 1 psychiatric disorder, and anxiety disorders are the most common disorder (7-9). In spite of the high prevalence of mental disorders, there is not enough service provision for their diagnosis and management. In 2006, WHO reported that 33 mental hospitals are available in Iran with a total of 5350 beds (7.9 per 100 000 populations). The number of psychiatrists, psychologists, and social workers were 1.2, 2, and 0.03 per 100 000 populations, respectively. However, this small scale of psychiatric and human resources is unequally divided in urban–rural areas and is mostly concentrated in large cities. The other problem in using psychiatric services is the stigma and self-stigma towards people with mental illness, so patients and their families are reluctant to visit psychiatric clinics. Moreover, follow-up visits can be time-consuming and costly for the patients and their families.

A few studies have used telepsychiatry service in Iran. Hajebi et al (2012) compared telephone with face-to-face interview for diagnosis of psychotic disorder in a clinical population. Their results showed that telephone interview was an effective method to differentiate between individuals with and without lifetime psychotic disorders (10). Najafi et al (2015) who conducted a study on a group of patients with ADHD found that telepsychiatry had a desirable effect on patient satisfaction and was cost-effective (11). 

Worldwide evidence exists about the benefits of telepsychiatry. However, there are many differences between countries in patients’ culture and telecommunication technologies, which highlights the necessity of repeating evaluation of a telepsychiatry system in different countries. There are still many open questions in telepsychiatry in Iran which may have different answers considering cultural backgrounds and social barriers. First, diagnostic agreement between online and face-to- face interview needs to be taken into consideration. Also, another question that needs to be addressed is that whether psychiatric patients are willing to sit behind a monitor and be interviewed and whether they could trust the system and provide reliable information. In this study, a telepsychiatry project was conducted to compare inpatient psychiatric diagnosis via interactive video with face-to-face interviews and to investigate patient and doctor satisfaction.

## Materials and Methods

***Participants***

This study was conducted in an inpatient department of a university-affiliated hospital in Kerman University of Medical Sciences, Iran (Shahid-Beheshti hospital). The study sample consisted of 40 inpatients aged over 18 years who were selected from October 2016 to February 2017. All patients were selected by a psychiatric resident (O.M.) after an interview to ensure they had no severe disorganized behavior, communication and language impairments, mental retardation, and sever agitation. All participants received their medications. The Research was approved by the ethics committee of Kerman University of Medical Sciences.

***Procedure***

The psychiatric resident prepared the setting and coordinated the procedure of data collection. He selected the patients, explained research processes, obtained written informed consent, and introduced patients to the interviewers. Two psychiatric consultants who were both deputy members of Kerman University of Medical Sciences conducted the interviews (S.M. and A.N.). Before collecting the data, diagnostic agreement was assessed by the 2 interviewers for 14 patients through face-to-face interviews; and the interviewers had acceptable diagnosis agreement.

All patients were interviewed by a psychiatrist once by face-to-face conventional method and once by interactive video teleconsultation by another psychiatrist using Skype software. A DSM-5 diagnosis was recorded by each psychiatrist for each patient. For the telepsychiatry session, prior to the patient’s interview, the psychiatric resident (O.M.) provided a brief history about the patient for the psychiatric consultant (S.M.) including patient’s name, age, number of hospitalizations, reasons of referral, starting time of symptoms, and a brief history of the present illness. Then, the consultant interviewed the patient for about 30 minutes and recorded the diagnosis. In another day, the second psychiatric consultant (A.G.) visited each patient, performed a face-to-face interview, and recorded the diagnosis. If a patient received more than one psychiatric diagnosis, the primary diagnosis was used for the analysis.

All technological issues regarding computers and telecommunication technologies were supervised by Information Technology (IT) Department of Shahid Beheshti hospital and any problems was managed by the IT team. 

At the end of telepsychiatry session, patients were asked to complete the 9-item Patient's Satisfaction Questionnaire, which assessed their attitude towards telepsychiatry visit. In this study, all the questions were used according to the study by Mucic et al, 2010, except for 1 question. Moreover, 2 more questions (questions 4 and 6) were added (Table 1). 12 The questionnaire assessed patient's satisfaction considering the following aspects: (1) information, (2) technology, (3) confidentiality, (4) preference, and (5) future attitudes. Finally, at the end of telepsychiatry visit, the consultant psychiatrist completed a 4-item questionnaire about suitability of patients for telepsychiatry visits (Table 2). 

***Data Analysis***

The agreement between the 2 psychiatrists was calculated with the kappa statistic, which accounts for an agreement that occurs by chance. Kappa estimates were considered as excellent (>0.92), good/very good (>0.6), and fair (>0.4) as suggested by Byrt.13 Data were analyzed using standard packages SPSS 17.

## Results

A total of 40 patients (males = 29) participated in this study. The average age of the participants was 35.2 years (rang = 21-62) and their mean years of education was 8.8 years (SD = 3.6). Results of this study showed that the diagnostic agreement between the 2 interviewers was 75%.

A total of 35 patients reported that the information about telepsychiatry was easy to understand. Also, almost all patients (39), except for 1, were satisfied with the quality of sound and picture. 

Moreover, 38 patients perceived the visits through telepsychiatry to be uncomfortable to some degree and 2 patients found telepsychiatry comfortable. Furthermore, 36 patients reported feeling safe during telepsychiatry, while 4 did not find it safe enough. Also, most of patients (33) reported worries about information disclosure to some degree. Also, 31 patients reported that they were able to explain their problems through telepsychiatry visit, while the rest of the sample (9) encountered some problems.

Four patients did not prefer to have telepsychiatry follow-up visits, whereas 34 patients had complete preference and 2 chose “in some degree” in the questionnaire. Five patients reported that they would not recommend telepsychiatry to others, while the rest of the sample stated that they would recommend it to others. 

The psychiatric consultant believed that 12 patients could not explain their problem well through telepsychiatry interview and needed a face-to-face interview. She agreed that telepsychiatry was an acceptable way for the majority of the patients (30). Also, the psychiatrist identified 19 patients who needed to have a family member with them at the time of the complementary interview. 

**Table 1:** Patient Satisfaction Questionnaire about Telepsychiatry

	**Yes, in high ** **degree** **N (%)**	**Yes, In ** **some ** **degree** **N (%)**	**No, only in ** **less degree** **N (%)**		**No, not at ** **all** **N (%)**	**Don’t ** **know** **N (%)**
1. Did you get enough information about telepsychiatry?	34(85)	0	0		6 (15)	0
2. Do you perceive contact via TV as uncomfortable?	0	38 (95)	0		2 (5)	0
3. Did you feel safe with telepsychiatry contact?	36 (90)	0	0		4 (10)	0
4. Did you worry about disclosure of your personal information?	1(2.5)	33 (82.5)	0		6 (15)	0
5. Could you express everything you wanted?	31(77.5)	0	0		9 (22.5)	0
6. Do you prefer telepsychiatry for follow-up visits?	34 (85)	2 (5)	0		4 (10)	0
7. Were you satisfied with sound quality?	39 (97.5)	0	0		1 (2.5)	0
8. Were you satisfied with picture quality?	39 (97.5)	0	0		1 (2.5)	0
9. Would you recommend telepsychiatry to others?	33 (82.5)	2 (5)	0		5 (12.5)	0

**Table 2:** Doctor Satisfaction Questionnaire about Telepsychiatry

	**Yes** **N (%)**	**No** **N (%)**
1. It is also required to visit patient in a face-to-face interview.	12 (30)	28 (70)
2. Did patients explain her/his problems as easy as face-to-face interview?	28 (70)	12 (30)
3. Is it required to interview with a family member?	19 (47.5)	21 (52.5)
4. Was Videoconference visit suitable for the patient?	30 (75)	10 (25)

## Discussion

Telepsychiatry is an important component of mental health, as it is an easily accessible and cost-effective psychiatric care. The present study assessed some aspects of telepsychiatry in Iran as a developing country with its cultural and social characteristics. 

Results of this study showed preliminary evidence of acceptable agreement between distant assessment through telepsychiatry and face-to-face assessment in Iran. The finding of this study is consistent with previous studies that compared the use of telepsychiatry to face-to-face evaluations.14-16 For example, Seidal et al (2014) found 86% agreement in an emergency department between psychiatrists, when one used face-to-face and one used telepsychiatry (17). A meta-analysis of 14 studies showed no difference in accuracy between telepsychiatry and face-to-face evaluations based on objective assessment instruments.18

User’s participation and satisfaction are important in ensuring that new technological methods are suitable for specific patients. Although a large body of studies have reported positive views of patients about telepsychiatry, 19, 20 it is necessary to assess it in every country and population, as each population has its own specific culture. Also, the results indicated that 85% of patients preferred to use telepsychiatry for follow-up visits. The study participants were inpatients that usually need long-term follow-ups. Therefore, by telepsychiatry, many trips between extreme distances could be reduced considering that these travels are made to receive an only 20-minute consultation. Decreasing the number of trips between cities results in saving time and reducing absence from work. Another important benefit is reducing financial burden on families (18, 21).

Consistent with previous studies, 12, 21 patients expressed a high degree of satisfaction with quality of sound and images, which indicates that using Skype can be a good candidate for such service in Iran. In this study, Skype was used as a telecommunication method for the telepsychiatry project. The authors were aware of some concerns for security and privacy of Skype; however, Skype Company declares that all conversations are encoded securely and users are ensured that their security and privacy are protected.22 Also, Skype is a user-friendly platform which works very well with Iran’s internet infrastructure and is also a free software that is easily accessible. Also, there is not any regulation in Iran against using Skype. Nevertheless, it would have been better to use video conference system, but it would have increased the cost of the project. This was a pilot project whose main aim was to explore the feasibility of a telepsychiatry consultation. To provide a long-term telehealth service, using a videoconference system would be much more acceptable compared to Skype in terms of security and privacy issues.

Most of patients reported that they were uncomfortable and worried about the safety of information to some degree. One explanation may be that the patients were not reassured that no one was listening, watching, or recording the session. This finding indicates that explaining the privacy of the setting and its confidentiality is highly important to the patients. Moreover, all participants were inpatients and other factors such as the patients’ educational level and severity of their mental illness might have contributed to these concerns. In 2010, Mucic proposed that severe mental disorders (eg, psychosis) and poor education level could reduce patient’s satisfaction, particularly in the case of perceived comfort and safety of information (12).

Telepsychiatry would be successful only if psychiatrists are satisfied with this service and offer it to their patients. In agreement with previous studies, 23-25 the consultant psychiatrist found that information obtained by telepsychiatry was similar to face-to-face interview. This evidence supports good cooperation of patients and usefulness of this method in Iran. 

This study had some limitations such as small sample size and absence of a standardized instrument for diagnosis. Moreover, in this study, the psychiatric resident was familiar with psychiatric patients and knew what aspects of patient’s history are important. However, in real situations usually a nurse or general practitioner or even a family member of the patient would introduce and helping patients remotely by the psychiatrists. Therefore, it would be highly important to have trained personnel to assist the patients during teleconsultation. Also, all participants were inpatients who usually have severe mental disorders such as schizophrenia, mood disorders, and other psychotic disorders. This might have contributed to the high diagnostic agreement of the present study. However, future research needs to examine diagnostic agreement in a sample of outpatients. 

This study showed that in an inpatient setting where there are serious psychiatric disorders, telepsychiatry and face-to-face assessment function similarly and both the patient and the provider have a positive opinion about telepsychiatry. This survey may encourage the policymakers to move toward implementing teleconsultation infrastructures and training and encouraging psychiatrists to use telepsychiatry.

## Limitation

Our main limitation was internet speed which caused some interruption during the interview with patients. 

## Conclusion

Telepsychiatry service can be used for psychiatric evaluation in Iran, and it has a desirable effect on patient and doctor satisfaction. The results of this study showed the capacity of moving towards using telepsychiatry this survey may encourage the policymakers to move toward implementing teleconsultation infrastructures and training and encouraging psychiatrists to use telepsychiatry.
